# Dimeth­yl(2,2′:6′,2′′-terpyridine-κ^3^ *N*,*N*′,*N*′′)zinc(II)

**DOI:** 10.1107/S1600536809040136

**Published:** 2009-10-07

**Authors:** Tamila Shalumova, Joseph M. Tanski

**Affiliations:** a Department of Chemistry, Vassar College, Poughkeepsie, NY 12604, USA

## Abstract

The title compound, [Zn(CH_3_)_2_(C_15_H_11_N_3_)], was synthesized by the addition of dimethyl­zinc to 2,2′:6′,2′′-terpyridine and was crystallized by the slow evaporation of THF. The penta­coordinate Zn^II^ atom, lying on a twofold rotation axis, displays a distorted trigonal-bipyramidal geometry, with two terminal N atoms at the axial positions and the central N atom and two methyl C atoms at the equatorial positions.

## Related literature

For the crystal structures of terpyridine dichlorido­zinc(II) compounds, see: Corbridge & Cox (1956 ▶); Einstein & Penfold (1966 ▶); Vlasse *et al.* (1983 ▶). For examples of other substituted terpyridine zinc(II) compounds, see: Harrison *et al.* (1986 ▶); Hou *et al.* (2004 ▶). The structure of a bipyridine dimethyl­zinc(II) compound was reported by Wissing *et al.* (1994 ▶).
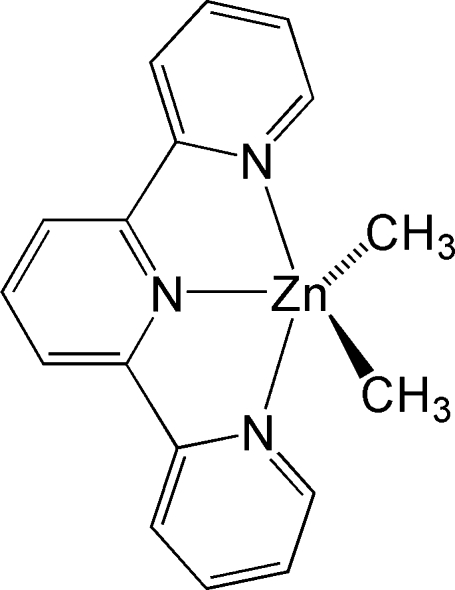


## Experimental

### 

#### Crystal data

[Zn(CH_3_)_2_(C_15_H_11_N_3_)]
*M* *_r_* = 328.71
Monoclinic, 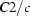

*a* = 17.4250 (11) Å
*b* = 9.1083 (6) Å
*c* = 11.7595 (14) Å
β = 127.193 (1)°
*V* = 1486.8 (2) Å^3^
*Z* = 4
Mo *K*α radiation
μ = 1.65 mm^−1^
*T* = 125 K
0.23 × 0.13 × 0.06 mm

#### Data collection

Bruker APEXII CCD diffractometer
Absorption correction: multi-scan (*SADABS*; Sheldrick, 1996 ▶) *T*_min_ = 0.703, *T*_max_ = 0.908
9483 measured reflections
1837 independent reflections
1710 reflections with *I* > 2σ(*I*)
*R*_int_ = 0.025

#### Refinement

*R*[*F* ^2^ > 2σ(*F* ^2^)] = 0.022
*wR*(*F* ^2^) = 0.061
*S* = 1.09
1837 reflections
98 parameters
H-atom parameters constrained
Δρ_max_ = 0.41 e Å^−3^
Δρ_min_ = −0.27 e Å^−3^

### 

Data collection: *APEX2* (Bruker, 2007 ▶); cell refinement: *SAINT* (Bruker, 2007 ▶); data reduction: *SAINT*; program(s) used to solve structure: *SHELXS97* (Sheldrick, 2008 ▶); program(s) used to refine structure: *SHELXL97* (Sheldrick, 2008 ▶); molecular graphics: *SHELXTL* (Sheldrick, 2008 ▶); software used to prepare material for publication: *SHELXTL*.

## Supplementary Material

Crystal structure: contains datablocks global, I. DOI: 10.1107/S1600536809040136/hy2233sup1.cif

Structure factors: contains datablocks I. DOI: 10.1107/S1600536809040136/hy2233Isup2.hkl

Additional supplementary materials:  crystallographic information; 3D view; checkCIF report

## Figures and Tables

**Table 1:** Selected bond lengths (Å)

Zn—C1	2.0282 (15)
Zn—N1	2.3381 (12)
Zn—N2	2.2603 (16)

**Fig. 1.**
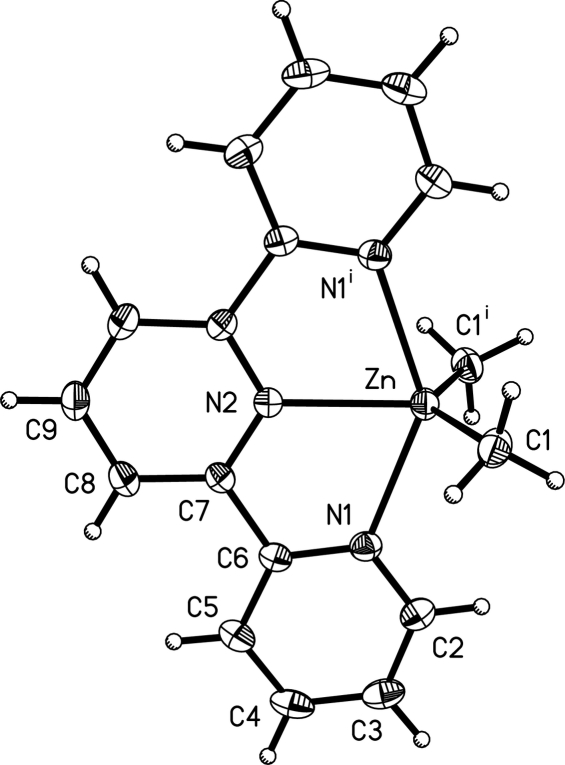
Molecular structure of the title compound, with displacement ellipsoids shown at the 50% probability level. [Symmetry code: (i) -x, y, 1/2-z.]

## supplementary crystallographic information

### Comment

The chelating ligand 2,2':6',2''-terpyridine coordinates to a variety of first-row transition metal Lewis acids. Specifically, it can coordinate to disubstituted zinc compounds, allowing for the formation of trigonal bipyramidal zinc(II) complexes (Harrison *et al.*, 1986). The structure of terpyridine dichlorozinc(II) has been reported a number of times with different results (Corbridge & Cox, 1956; Einstein & Penfold, 1966; Vlasse *et al.*, 1983). In addition to substituents on the zinc(II) center, substituents on the terpyridine are also known (Hou *et al.*, 2004). The structure presented here is the first known structure in a class of terpyridine zinc(II) compounds with two alkyl groups bound directly to the metal center.

The title compound (Fig. 1) was obtained by the reaction of dimethylzinc with 2,2':6',2''-terpyridine. It exhibits a distorted trigonal bipyramidal geometry about the metal center and has the two terminal N atoms in the axial positions, with a bond length of 2.3381 (12) Å (Table 1). The central N atom, coordinated to the zinc *via* the equatorial position, has a slightly smaller bond length, 2.2603 (16) Å, due to the size of the ligand, which is not able to wrap around the metal 180°. The N1—Zn—N1^i^ bond angle is 140.52 (6)° [symmetry code: (i) -x, y, 1/2-z], illustrating the degree to which the compound is distorted from a perfectly trigonal bipyramid. The Zn—C bond length is 2.0282 (15) Å, and the C1—Zn—C1^i^ bond angle is 133.21 (9)°: slightly greater than the expected 120° between equatorial atoms. Interestingly, the Zn—N bond lengths shown here are about 0.2 Å longer than those reported for similar terpyridine zinc(II) complexes, while the Zn—C length is expectedly shorter than the Zn—Cl and Zn—S lengths (Harrison *et al.*, 1986; Hou *et al.*, 2004; Vlasse *et al.*, 1983). However, the Zn—C bond length is similar to that reported for a bipyridine dimethylzinc(II) complex, characterized by Wessing *et al.* (1994). The terpyridyl N1—Zn—N1^i^ and N1—Zn—N2 bond angles are in agreement with those reported in the literature (Harrison *et al.*, 1986; Hou *et al.*, 2004; Vlasse *et al.*, 1983).

### Experimental

Under a nitrogen atmosphere, dimethylzinc (2 *M* in toluene, 1.07 ml, 2.14 mmol) was added to a stirring solution of terpyridine (0.511 g, 2.19 mmol) in toluene (3.5 ml). The resulting orange precipitate, which is extremely sensitive to hydrolysis, was filtered and dried *in vacuo* (yield 65%, 0.46 g). Crystallization was achieved by slow evaporation of THF in a nitrogen filled glovebox, which yielded yellow plates within five days. Analysis, calculated for C_17_H_17_N_3_Zn: C 62.11, H 5.21, N 12.78%; found: C 59.18, H 5.09, N 12.12%. ^1^H NMR (300 MHz, C_6_D_6_): δ -0.529 (s, 6H, –C*H*_3_).

### Refinement

A suitable crystal was mounted in a nylon loop with Paratone-*N* cryoprotectant oil and data was collected on a Bruker APEXII CCD platform diffractometer. H atoms were included in calculated positions and were refined using a riding model, with C—H = 0.95 (aromatic) and 0.98 (methyl) Å and with *U*_iso_(H) = 1.2(1.5 for methyl)*U*_eq_(C).

### Crystal data

**Table:**

[Zn(CH_3_)_2_(C_15_H_11_N_3_)]	*F*(000) = 680
*M**_r_* = 328.71	*D*_x_ = 1.469 Mg m^−^^3^
Monoclinic, *C*2/*c*	Mo *K*α radiation, λ = 0.71073 Å
Hall symbol: -C 2yc	Cell parameters from 6987 reflections
*a* = 17.4250 (11) Å	θ = 2.7–28.3°
*b* = 9.1083 (6) Å	µ = 1.65 mm^−^^1^
*c* = 11.7595 (14) Å	*T* = 125 K
β = 127.193 (1)°	Plate, yellow
*V* = 1486.8 (2) Å^3^	0.23 × 0.13 × 0.06 mm
*Z* = 4	

### Data collection

**Table:**

Bruker APEXII CCD diffractometer	1837 independent reflections
Radiation source: fine-focus sealed tube	1710 reflections with *I* > 2σ(*I*)
graphite	*R*_int_ = 0.025
φ and ω scans	θ_max_ = 28.3°, θ_min_ = 2.7°
Absorption correction: multi-scan (*SADABS*; Sheldrick, 1996)	*h* = −23→23
*T*_min_ = 0.703, *T*_max_ = 0.908	*k* = −12→12
9483 measured reflections	*l* = −15→15

### Refinement

**Table:**

Refinement on *F*^2^	Primary atom site location: structure-invariant direct methods
Least-squares matrix: full	Secondary atom site location: difference Fourier map
*R*[*F*^2^ > 2σ(*F*^2^)] = 0.022	Hydrogen site location: inferred from neighbouring sites
*wR*(*F*^2^) = 0.061	H-atom parameters constrained
*S* = 1.09	*w* = 1/[σ^2^(*F*_o_^2^) + (0.0347*P*)^2^ + 0.7204*P*] where *P* = (*F*_o_^2^ + 2*F*_c_^2^)/3
1837 reflections	(Δ/σ)_max_ < 0.001
98 parameters	Δρ_max_ = 0.41 e Å^−^^3^
0 restraints	Δρ_min_ = −0.27 e Å^−^^3^

### Fractional atomic coordinates and isotropic or equivalent isotropic displacement parameters (Å^2^)

**Table:**

	*x*	*y*	*z*	*U*_iso_*/*U*_eq_	
Zn	0.0000	0.27994 (2)	0.2500	0.02138 (9)	
N1	0.09270 (8)	0.19322 (13)	0.18122 (12)	0.0217 (2)	
N2	0.0000	0.03178 (17)	0.2500	0.0181 (3)	
C1	0.11086 (11)	0.36835 (17)	0.43838 (16)	0.0282 (3)	
H1A	0.1292	0.4625	0.4207	0.042*	
H1B	0.1660	0.3013	0.4858	0.042*	
H1C	0.0909	0.3840	0.4996	0.042*	
C2	0.13813 (11)	0.28148 (17)	0.14866 (16)	0.0260 (3)	
H2A	0.1337	0.3846	0.1557	0.031*	
C3	0.19141 (11)	0.22995 (19)	0.10513 (17)	0.0291 (3)	
H3A	0.2237	0.2959	0.0845	0.035*	
C4	0.19631 (10)	0.0801 (2)	0.09259 (16)	0.0298 (3)	
H4A	0.2317	0.0413	0.0621	0.036*	
C5	0.14921 (10)	−0.01296 (17)	0.12488 (15)	0.0261 (3)	
H5A	0.1513	−0.1163	0.1160	0.031*	
C6	0.09862 (9)	0.04762 (15)	0.17079 (13)	0.0197 (3)	
C7	0.04737 (9)	−0.04323 (15)	0.21108 (13)	0.0188 (3)	
C8	0.04827 (10)	−0.19665 (15)	0.20904 (15)	0.0239 (3)	
H8A	0.0814	−0.2475	0.1801	0.029*	
C9	0.0000	−0.2732 (2)	0.2500	0.0260 (4)	
H9A	0.0000	−0.3775	0.2500	0.031*	

### Atomic displacement parameters (Å^2^)

**Table:**

	*U*^11^	*U*^22^	*U*^33^	*U*^12^	*U*^13^	*U*^23^
Zn	0.02557 (13)	0.01758 (13)	0.02424 (13)	0.000	0.01676 (11)	0.000
N1	0.0219 (5)	0.0236 (6)	0.0222 (6)	−0.0008 (4)	0.0147 (5)	−0.0004 (4)
N2	0.0182 (7)	0.0178 (7)	0.0176 (7)	0.000	0.0105 (6)	0.000
C1	0.0330 (7)	0.0234 (7)	0.0303 (7)	−0.0055 (6)	0.0202 (7)	−0.0032 (6)
C2	0.0259 (7)	0.0279 (7)	0.0261 (7)	−0.0032 (6)	0.0167 (6)	0.0009 (6)
C3	0.0230 (7)	0.0420 (9)	0.0243 (7)	−0.0018 (6)	0.0154 (6)	0.0048 (6)
C4	0.0236 (7)	0.0461 (9)	0.0247 (7)	0.0082 (6)	0.0173 (6)	0.0050 (6)
C5	0.0252 (7)	0.0299 (8)	0.0253 (7)	0.0066 (6)	0.0164 (6)	0.0020 (6)
C6	0.0172 (6)	0.0242 (7)	0.0161 (6)	0.0021 (5)	0.0093 (5)	0.0006 (5)
C7	0.0177 (6)	0.0195 (6)	0.0167 (6)	0.0016 (5)	0.0091 (5)	−0.0005 (5)
C8	0.0230 (7)	0.0208 (7)	0.0237 (7)	0.0031 (5)	0.0119 (6)	−0.0023 (5)
C9	0.0262 (10)	0.0171 (9)	0.0276 (10)	0.000	0.0126 (9)	0.000

### Geometric parameters (Å, °)

**Table:**

Zn—C1^i^	2.0282 (15)	C2—H2A	0.9500
Zn—C1	2.0282 (15)	C3—C4	1.381 (2)
Zn—N1	2.3381 (12)	C3—H3A	0.9500
Zn—N2	2.2603 (16)	C4—C5	1.383 (2)
Zn—N1^i^	2.3382 (12)	C4—H4A	0.9500
N1—C2	1.3364 (19)	C5—C6	1.3958 (18)
N1—C6	1.3416 (18)	C5—H5A	0.9500
N2—C7^i^	1.3470 (15)	C6—C7	1.4893 (18)
N2—C7	1.3470 (15)	C7—C8	1.3979 (19)
C1—H1A	0.9800	C8—C9	1.3838 (18)
C1—H1B	0.9800	C8—H8A	0.9500
C1—H1C	0.9800	C9—C8^i^	1.3838 (18)
C2—C3	1.385 (2)	C9—H9A	0.9500
			
C1^i^—Zn—C1	133.21 (9)	N1—C2—H2A	118.4
C1^i^—Zn—N2	113.39 (5)	C3—C2—H2A	118.4
C1—Zn—N2	113.39 (5)	C4—C3—C2	118.22 (14)
C1^i^—Zn—N1	99.05 (5)	C4—C3—H3A	120.9
C1—Zn—N1	96.37 (5)	C2—C3—H3A	120.9
N2—Zn—N1	70.26 (3)	C3—C4—C5	119.41 (14)
C1^i^—Zn—N1^i^	96.37 (5)	C3—C4—H4A	120.3
C1—Zn—N1^i^	99.05 (5)	C5—C4—H4A	120.3
N2—Zn—N1^i^	70.26 (3)	C4—C5—C6	118.83 (14)
N1—Zn—N1^i^	140.52 (6)	C4—C5—H5A	120.6
C2—N1—C6	118.48 (12)	C6—C5—H5A	120.6
C2—N1—Zn	123.28 (10)	N1—C6—C5	121.84 (13)
C6—N1—Zn	118.22 (9)	N1—C6—C7	115.22 (11)
C7^i^—N2—C7	119.05 (16)	C5—C6—C7	122.93 (13)
C7^i^—N2—Zn	120.48 (8)	N2—C7—C8	121.89 (13)
C7—N2—Zn	120.47 (8)	N2—C7—C6	115.77 (12)
Zn—C1—H1A	109.5	C8—C7—C6	122.34 (12)
Zn—C1—H1B	109.5	C9—C8—C7	118.82 (14)
H1A—C1—H1B	109.5	C9—C8—H8A	120.6
Zn—C1—H1C	109.5	C7—C8—H8A	120.6
H1A—C1—H1C	109.5	C8—C9—C8^i^	119.53 (19)
H1B—C1—H1C	109.5	C8—C9—H9A	120.2
N1—C2—C3	123.19 (14)	C8^i^—C9—H9A	120.2
			
C1^i^—Zn—N1—C2	68.69 (12)	C2—C3—C4—C5	−0.6 (2)
C1—Zn—N1—C2	−66.99 (12)	C3—C4—C5—C6	−0.6 (2)
N2—Zn—N1—C2	−179.60 (12)	C2—N1—C6—C5	−1.1 (2)
N1^i^—Zn—N1—C2	−179.60 (12)	Zn—N1—C6—C5	177.37 (10)
C1^i^—Zn—N1—C6	−109.70 (11)	C2—N1—C6—C7	179.08 (12)
C1—Zn—N1—C6	114.62 (11)	Zn—N1—C6—C7	−2.45 (15)
N2—Zn—N1—C6	2.01 (9)	C4—C5—C6—N1	1.5 (2)
N1^i^—Zn—N1—C6	2.01 (9)	C4—C5—C6—C7	−178.65 (12)
C1^i^—Zn—N2—C7^i^	−89.74 (8)	C7^i^—N2—C7—C8	0.42 (9)
C1—Zn—N2—C7^i^	90.26 (8)	Zn—N2—C7—C8	−179.58 (9)
N1—Zn—N2—C7^i^	178.72 (7)	C7^i^—N2—C7—C6	−179.48 (12)
N1^i^—Zn—N2—C7^i^	−1.28 (7)	Zn—N2—C7—C6	0.52 (12)
C1^i^—Zn—N2—C7	90.26 (8)	N1—C6—C7—N2	1.31 (16)
C1—Zn—N2—C7	−89.74 (8)	C5—C6—C7—N2	−178.51 (11)
N1—Zn—N2—C7	−1.28 (7)	N1—C6—C7—C8	−178.59 (13)
N1^i^—Zn—N2—C7	178.72 (7)	C5—C6—C7—C8	1.6 (2)
C6—N1—C2—C3	−0.3 (2)	N2—C7—C8—C9	−0.83 (19)
Zn—N1—C2—C3	−178.64 (11)	C6—C7—C8—C9	179.07 (10)
N1—C2—C3—C4	1.1 (2)	C7—C8—C9—C8^i^	0.39 (9)

Symmetry codes: (i) −*x*, *y*, −*z*+1/2.

## References

[bb1] Bruker (2007). *APEX2* and *SAINT* Bruker AXS Inc., Madison, Wisconsin, USA.

[bb2] Corbridge, D. E. C. & Cox, E. G. (1956). *J. Chem. Soc.* pp. 594–603.

[bb3] Einstein, F. W. B. & Penfold, B. R. (1966). *Acta Cryst.* **20**, 924–926.

[bb4] Harrison, P. G., Begley, M. J., Kikabhai, T. & Killer, F. (1986). *J. Chem. Soc. Dalton Trans.* pp. 929–938.

[bb5] Hou, L., Li, D. & Ng, S. W. (2004). *Acta Cryst.* E**60**, m1734–m1735.

[bb6] Sheldrick, G. M. (1996). *SADABS* University of Göttingen, Germany.

[bb7] Sheldrick, G. M. (2008). *Acta Cryst.* A**64**, 112–122. 10.1107/S0108767307043930 18156677

[bb8] Vlasse, M., Rojo, T. & Beltran-Porter, D. (1983). *Acta Cryst.* C**39**, 560–563.

[bb9] Wissing, E., Kaupp, M., Boersma, J., Spek, A. L. & van Koten, G. (1994). *Organometallics*, **13**, 2349–2356.

